# Somatic embryogenesis and shoot organogenesis in peanut cv. ‘Georgia-12Y’ and successful transfer to the soil

**DOI:** 10.1371/journal.pone.0315060

**Published:** 2024-12-06

**Authors:** Poonam Khatri, Nirmal Joshee

**Affiliations:** Agricultural Research Station, Fort Valley State University, Fort Valley, Georgia, United States of America; Ataturk University: Ataturk Universitesi, TÜRKIYE

## Abstract

An efficient regeneration system was established through somatic embryogenesis and shoot organogenesis using mature embryos explants of peanut cultivar ‘Georgia-12Y’. The role of plant growth regulator combinations was investigated for embryogenic callus and somatic embryo induction. Results showed that Murashige and Skoog (MS) medium supplemented with 20 μM picloram (4-amino 3, 5, 6-trichloropicolinic acid), casein hydrolysate (0.2 g/L), sucrose (30 g/L) and sorbitol (10 g/L) supported callus induction in dark and higher number of somatic embryos in light. No somatic embryos were induced at 0.1 μM to 10.0 μM of 2,4-Dichloro phenoxy acetic acid (2,4-D) and picloram individually. The highest regeneration frequency of 90% was recorded on 40 μM 2,4-D + casein hydrolysate (0.2 g/L), sucrose (30 g/L) and sorbitol (10 g/L). The plantlets regenerated via somatic embryogenesis did not exhibit any morphological abnormalities. Double staining with acetocarmine and Evans blue distinguished between embryogenic and non-embryogenic callus. Histological observations confirmed distinct developmental stages of somatic embryos. On the other hand, highest number of shoots were induced in response to MS + 15 μM thidiazuron (TDZ) among various treatments tested. Incubation of shoots on plant growth regulator free MS medium induced *in-vitro* flowering after 12 weeks under light conditions. The induction of embryogenic and morphogenic callus and production of fertile peanut plants using manipulations of various plant growth regulators is reported on peanut cultivar ‘Georgia- 12Y’.

## Introduction

Peanut (*Arachis hypogaea* L.) belongs to the genus *Arachis* which has 9 intrageneric taxonomic sections based on morphology, cross compatibility, and geographic distribution [1]. It is the choicest legume crop in the world rich in proteins, iron, calcium, vitamin B complex (riboflavin, niacin, and thiamine) and different fatty acids for human and livestock consumption [2]. Georgia-12Y is a runner type peanut cultivar and commercially popular in the Southeastern United States (https://extension.uga.edu/publications/series/detail/90/2024-georgia-ag-forecast.html). Georgia-12Y is a high-yielding, medium-seeded, runner-type peanut (*Arachis hypogaea L*. *subsp*. *hypogaea var*. *hypogaea*) cultivar that performed well in multi-locational trials and possess additional agronomic traits like resistance to tomato spotted wilt virus and white mold or stem rot (caused by *Sclerotium rolfsii* Sacc.). Lately, plant derived human vaccines are gaining importance, and peanut based vaccines have been found effective against *Helicobacter pylori* infection in humans [3], *Rinderpest* virus (cattle plague disease) in cattle and *Peste des petits ruminant virus* (PPRV) in sheep [4]. However, peanut production and quality are significantly affected by adverse climatic conditions, diseases, and pests. Therefore, an efficient *in vitro* regeneration system is a prerequisite for the conservation of germplasm and the improvement of plants with important agronomic traits employing various biotechnological techniques [5].

Somatic embryogenesis is known as the most appropriate *in vitro* system for the mass multiplication and genetic improvement of several legumes [6]. In somatic embryogenesis, somatic cells undergo a series of morphological and biochemical transformation to become embryogenic cells and eventually developing into a somatic embryo under suitable inductive conditions [6]. Somatic embryogenesis provides an option for studying developmental processes involved in regeneration, organogenesis, and developmental embryology under *in-vitro* conditions and somatic embryos can be stored and observed for molecular and biochemical analysis [7]. Significant efforts have been made to enhance the regeneration frequency in peanuts, but it is still difficult to get enough number of explants in a short duration [8]. There are many studies reported on *in vitro* regeneration in peanuts (Table 1). In addition, low frequency embryo induction, poor germination, low conversion of somatic embryos into plantlets and most importantly genotype specificity are the major limitations of somatic embryogenesis in legumes [9]. Because of genotypic specificity, cultural environment and media often need to be varied from one genus or species of plant to another [10]. Also, before initiating a peanut improvement program using *in-vitro* techniques, it is crucial to assess the morphogenic potential of the existing cultivars [11]. The choice of explant and the supplementation of plant growth regulator(s) to the culture medium are important factors in the induction of somatic embryos. A mature zygotic embryo as an explant may overcome many challenges associated with immature zygotic embryos and other immature explants used in peanut micropropagation. Mature zygotic embryos are available all-round the year and, no greenhouse or field planting is required resulting in less chances of contamination [12]. Auxins are used to initiate and regulate somatic embryogenesis, mainly by promoting callus formation, regulating cell division, and developing embryos [13]. Among all the Auxin 2, 4-D has been frequently used to induce somatic embryogenesis in many plants, but higher concentrations may induce somaclonal variation [13]. Therefore, investigating the use of other auxins at low concentrations for inducing somatic embryogenesis would be useful.

**Table 1:** A review of *in vitro* propagation research in *Arachis hypogaea* L.

S. No.	Explants	Somatic Embryogenesis	Shoot Initiation	Peanut type	References
1	Cotyledon	10 mg/L 2,4-D		*A*. *hypogaea* L.cv.Heiyuzhen	[14]
2	Immature cotyledon	90.4 μM 2,4-D	30 mg/L BAP	cv JLM-1 and J11 (Indian)	[15, 16]
3	Mature and immature Cotyledon, Embryo axes, Epicotyl, Mature and immature embryo, young leaflets, Leaflet segments	2, 4-D, TDZ, NAA, BAP, picloram were tested and 3–7 mg/L	4.0 mg/L BAP	Runner, Virginia, Valencia and Spanish	[17]
			+ 2.0 mg/LNAA		
		2, 4-D showed the best response			
4	Mature, dry epicotyls	83.0 μM and 124.4 μM centrophenoxine	-	Runner and Virginia	[18]
5	Cotyledonary nodes	19 mg/L picloram,	-	Runner	[19, 20]
		0.5 mg/L NAA,			
		5.0 mg/L BAP			
6	Mature embryo axes	5–30 mg/L 2,4-D		*A*. *hypogaea* L. (Serenut 4 T, Serenut 1R and Acholi-white)	[21]
7	Leaf discs	-	0.5 mg/L NAA + 0.5	Spanish and Baisha1016 (Chinese cultivar)	[5, 22]
			mg/L TDZ		
			8 mg/L BAP + 0.5		
			mg/L NAA		
8	Cotyledonary node	-	5.0 mg/l BAP	Virginia	[23]
9	Embryonic leaflets	10 mg/L 2, 4-D	4.0 mg/L BAP	Huayu 20, 22, 23, 25, and Luhua	[24]
10	Mature zygotic embryo derived leaflets	90.5 μM 2,4-D	17.83 μM NAA and	Runner and Virginia	[11]
			22.19 μM BAP		

2,4-D: 2,4-Dichlorophenoxyacetic acid, NAA: 1-Naphthalene acetic acid, BAP: 6-Benzyleaminopurine

TDZ: Thidiazuron.

The present study includes the effect of various auxins, cytokinin, sucrose, pH, culture media and gelling agents on mature zygotic embryo explant for somatic embryogenesis in the runner cultivar ‘G-12Y’ of peanut (Table 2). Given the low frequency of embryo induction, poor germination, low plantlet conversion, and genotypic specificity, the present investigation on *in-vitro* plant regeneration for peanut cultivar ‘G-12Y’was conducted. This study aims to provide a foundation for future genetic transformation, breeding efforts, and to study plant—pathogen interaction in peanut.

**Table 2:** Somatic embryo induction in response to various plant growth regulators in peanut ‘G-12Y’ mature embryo explants.

Treatments	Medium	BAP (μM)	2,4-D (μM)	Picloram (μM)	Sucrose (g/L)	Casein hydroly-sate (g/L)	Sorbitol (g/L)	Gelling agent (g/L)	pH	SE Induction
Control	MS	-	-	-	30	-	-	Agar (7.0)	5.8	No response
1	MS	2.5	5.0	-	20	-	-	Agar (7.0)	6.0	Callus
2	MS	5.0	2.5	5.0	20	-	-	Agar (7.0)	6.0	*Somatic embryos*
3	N&N	8.9	-	4.5	3.0	-	-	Phytagel (3.0)	5.7	*Somatic embryos*
4	MS	-	20.0	-	3.0	0.2	-	Agar (7.0)	5.8	Callus
5	MS	-	30.0	-	3.0	0.2	-	Agar (7.0)	5.8	Callus
6	MS	-	40.0	-	3.0	0.2	10	Agar (7.0)	5.8	*Somatic embryos*
7	MS	-	-	20.0	3.0	0.2	10	Agar (7.0)	5.8	*Somatic embryos*
8	MS	-	-	30.0	3.0	0.2	10	Agar (7.0)	5.8	Callus
9	MS	-	**-**	40.0	3.0	0.2	10	Agar (7.0)	5.8	Callus
10	MS	-	0.1		3.0	-	-	Agar (7.0)	5.8	Elongation of embryos
11	MS	-	1.0		3.0	-	-	Agar (7.0)	5.8	Elongation of embryos
12	MS	-	10.0		3.0	-	-	Agar (7.0)	5.8	Callus
13	MS	-	-	0.1	3.0	-	-	Agar (7.0)	5.8	Callus
14	MS	-	-	1.0	3.0	-	-	Agar (7.0)	5.8	Callus
15	MS	-	-	10.0	3.0	-	-	Agar (7.0)	5.8	Callus
16	MS	-	0.05	0.05	3.0	-	-	Agar (7.0)	5.8	Elongation of embryos
17	MS	-	0.5	0.5	3.0	-	-	Agar (7.0)	5.8	Elongation of embryos
18	MS	-	5.0	5.0	3.0	-	-	Agar (7.0)	5.8	*Somatic embryos*

SE: Somatic Embryo; N&N: Nitsch and Nitsch

## Materials and methods

### Plant materials

Mature certified seeds of peanut cultivar ‘Georgia- 12Y’ (Birdsong Peanuts, Blakely, GA, USA) were collected and rinsed under running water for 10 minutes, followed by an additional 45 minutes in distilled water containing 2–3 drops of Tween-20 (Sigma, MO) and 2% Fungigone^™^ (Plant media, USA) with constant stirring. After 45 min seeds were washed with distilled water for 10 min and then transferred to the laminar air flow cabinet. Washed seeds were dipped in 70% ethanol for 1 min and followed by two rinses of autoclaved deionized water. At this point remaining seed coats were removed and the embryos were excised after splitting cotyledons. Extracted embryos were surface sterilized by immersing them in 15% commercial bleach Clorox (Oakland, California, USA) for 15 min with continuous stirring followed by three 5 min washes in sterile distilled water [25].

### Culture conditions and media preparation for the callus induction and somatic embryo formation

Murashige and Skoog, 1962 (MS) and Nitsch and Nitsch, 1969 (Phytotechnology Labs, Kansas, USA) were used as basal media supplemented with 3.0% and 2.0% sucrose as the carbon source [26, 27]. Agar (0.7%, Phytotechnology Labs, Kansas, USA) and phytagel (0.3%, Phytotechnology Labs, Kansas, USA) were used as solidifying agents. The pH of the medium was adjusted to 5.8–6.0 before adding the gelling agents (agar and phytagel) and plant growth regulators to the medium. Ten embryos were inoculated in each Fisherbrand petri dish (90 x 15 mm; Fisher Scientific, USA), and replicated three times for each treatment. All the cultures were incubated in the dark for 4 weeks for callus induction. Various concentrations of auxins and cytokinins along with different concentrations of sucrose and gelling agents were studied to investigate their role in somatic embryogenesis (Table 2).

### Media preparation and culture conditions for the induction and elongation of shoots

MS basal medium was supplemented with four concentrations of 1, 5, 10 and 15 μM TDZ for the shoot induction and 5 μM BAP with 1.0 μM Gibberellic acid for shoot elongation. Sucrose (3.0%) and TC Agar (0.7%) were supplemented as the carbon source and gelling agent, respectively. The pH of the medium was adjusted to 5.8–6.0 before adding plant growth regulators and agar. For a control, MS medium lacking plant growth regulators was used. Twenty-five mL culture medium was dispensed in Fisherbrand petri dishes (90 x 15 mm; Fisher Scientific, USA). Cultures were incubated at 25 ± 2°C under 16 h light at 60 μ mol m^-2^ s^-1^ intensity. Shoots induced on shoot induction medium were transferred to shoot elongation medium after 4 weeks. Observations on the progress of cultures were recorded every week and the final data on the number of shoots per explants was calculated after 8 weeks (Table 3).

**Table 3:** Effect of plant growth regulators on adventitious shoots induction from mature embryo explants in peanut ‘G-12Y’ after 8 weeks.

Treatments	Shoot elongation medium, MS Medium + PGR (μM)			Number of shoots elongated per explants (Mean ± SE)
	BAP	GA_3_	TDZ	
Control	-	-	-	0.00 ± 0.00^c^
1	5	1	1	1.93 ± 0.33^b^
2	5	1	5	2.00 ± 0.23^b^
3	5	1	10	2.53 ± 0.30^b^
4	5	1	15	**5.40 ± 0.51** ^**a**^

Each value is expressed as Mean ± SE. Values with the same superscript are not significantly different at the 5% probability level, as determined by DMRT. Control treatments were devoid of growth regulators. BAP: 6-Benzyl aminopurine, TDZ: Thidiazuron and GA_3_: Gibberellic acid.

### Cytochemical analysis of embryogenic callus

Acetocarmine and Evans blue staining was used to differentiate embryogenic (ECs) and non-embryogenic calli (non-ECs) [28]. Five-week-old callus was used for differentiating embryogenic and non-embryogenic cells. Growing calli (2–5 mm in diameter) were collected from the petri dishes inoculated for embryogenesis and a piece was placed on a slide. Callus mass was gently pressed to release clumps of cells and then stained with 2% acetocarmine for 60 seconds (depending upon callus size) until the callus was fully soaked. Then, the overstained callus was gently rinsed 2–3 times with distilled water to wash away excess stain. After washing, acetocarmine-stained cells were further stained with 0.5% (w/v) Evans blue for 30 seconds and then washed two times with distilled water to remove non-specifically bound stain. Double-stained cells were placed on a slide with 1–2 drops of glycerol and were mounted with the coverslip for microscopic observation. Stained callus cells were observed under Olympus BX 43 microscope (Olympus, Bartlett, TN, USA).

### Acclimatization, hardening, and greenhouse transfer of plants

The plants regenerated through somatic embryogenesis were removed gently from the culture vessel and then roots were carefully washed to eliminate any remaining media. Washed plants were transferred into humidity-controlled acclimatization boxes (Smithers-Oasis, Kent, OH, USA) containing autoclaved Promix BX (Premier Horticulture Inc., Quakertown, PA, USA). These plants were incubated at 25 ± 2°C under diffused light for about two weeks. The acclimatization boxes contain a humidity control filter assisted with a wheel regulating humidity exchange. Filter was kept closed for the first two weeks to maintain 100% humidity. After two weeks, the humidity was reduced by gradually opening the wheel every day for one week. Once the hardened plants with new growth were observed, plants were transferred to larger pots with a perlite and potting mix (1:3 ratio), moistened with tap water and maintained in the greenhouse for further growth at 65–70% relative humidity and 27 ± 3°C.

### Histological observations

Nodular embryogenic callus and somatic embryos harvested at various developmental stages were fixed in FAA (formaldehyde 37%: glacial acetic acid: 100% ethanol: distilled water; 10:5:50:35) for 24 hours in a baby food jar. Dehydration and infiltration steps were carried out in an ascending series of Isopropyl alcohol (IPA) (Thermo Scientific, MA, USA) using 30, 50, 70, 90, and 100% concentrations for 45 min each at 65°C. For infiltration, paraffin wax Type 9 (Epredia^™^, USA) was added to the jars containing samples and IPA and incubated at 65°C in an oven. The infiltrated samples were then embedded in paraffin using Microtome EC 350 embedding machine (Thermo Scientific, MA, USA) in a cassette (Unisette^™^ cassette; Ted Pella, Inc., CA, USA) and 10 μm thick sections were cut using a Microm HM 355-S microtome (Thermo Fisher Scientific, MA, USA). The paraffin ribbons containing sections were then placed in a water bath for stretching (Tissue Prep TM Floatation Bath; Fisher Scientific, MA, USA) at 43°C. Stretched sections were placed on the slides and dried overnight at 40°C using a slide warmer (Triangle Biomedical Sciences, NC, USA). Slides were then deparaffinized by three successive washes of Xylene for 5 min each. Deparaffinized sections were stained with a few drops of Toluidine blue O stain (0.1%). The excess stain from the section was removed by a distilled water rinse and then covered by a few drops of acrytol mounting medium (Leica Biosystems, MA, USA) and glass coverslip before visualizing under the microscope (Olympus BX 43, Olympus, Bartlett, TN, USA) [29].

### Statistical analysis

The evaluated parameters were statistically processed using analysis of variance (ANOVA) and compared means by Duncan’s Multiple Range Test (DMRT) at a 0.05 probability level, with SPSS statistical software [IBM SPSS Statistics version 22.0 (stats.exe)]. The results were shown as mean ± S.E. of the triplicates. Experiments for somatic embryogenesis and shoot organogenesis were set up with 15 and 30 explants per treatment, respectively and each experiment was repeated three times.

## Results

### Callus induction and somatic embryos from mature embryo

Eighteen treatments based on MS and Nitsch and Nitsch media were evaluated for callus induction and somatic embryogenesis in peanut cultivar ‘G- 12Y’. Individual and low concentrations (0.1 μM to 10.0 μM) of growth regulators caused elongation of embryo explants and lack of embryogenic callus, eventually resulting browning of the explants (Table 2). It was observed that various combinations of auxins and cytokinin along with various pH, sucrose, and additives on MS and Nitsch and Nitsch media had a positive influence on somatic embryogenesis (Table 2).

**Fig 1:**
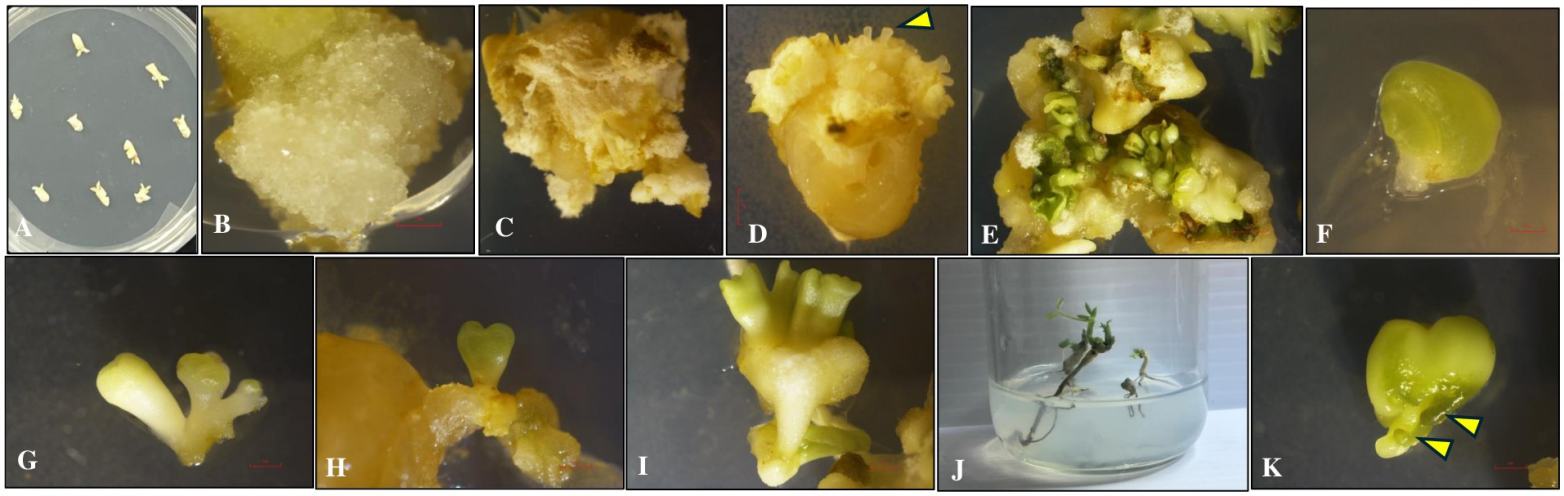
Somatic embryogenesis in peanut cultivar ‘G-12Y’. (A) Mature embryo explants cultured on callus induction medium (1^st^ Day). (B) Callus induction on mature embryo explant (MS medium + 1μM 2,4-D) in dark after 1 week. (C) Friable embryogenic callus on mature embryo explant (MS medium + 20μM picloram) in dark after 4 weeks. (D) Somatic embryos induced (yellow arrow) on MS medium with 20 μM picloram (dark) after 3 weeks. (E) Green somatic embryos on different developmental stages after 5 weeks (light) on PGR free MS medium. (F) Fasciated somatic embryo on PGR free MS medium (Light) after 7 weeks. (G-I) Developmental stages during somatic embryogenesis on PGR free MS medium (Light). (J) An isolated germinating somatic embryo with shoot and root formation on PGR free MS medium. (K) Secondary somatic embryos (yellow arrow) on PGR free MS Medium (Light). The diameter of the culture jar is 60 mm. Bars = 2 mm.

**Fig 2:**
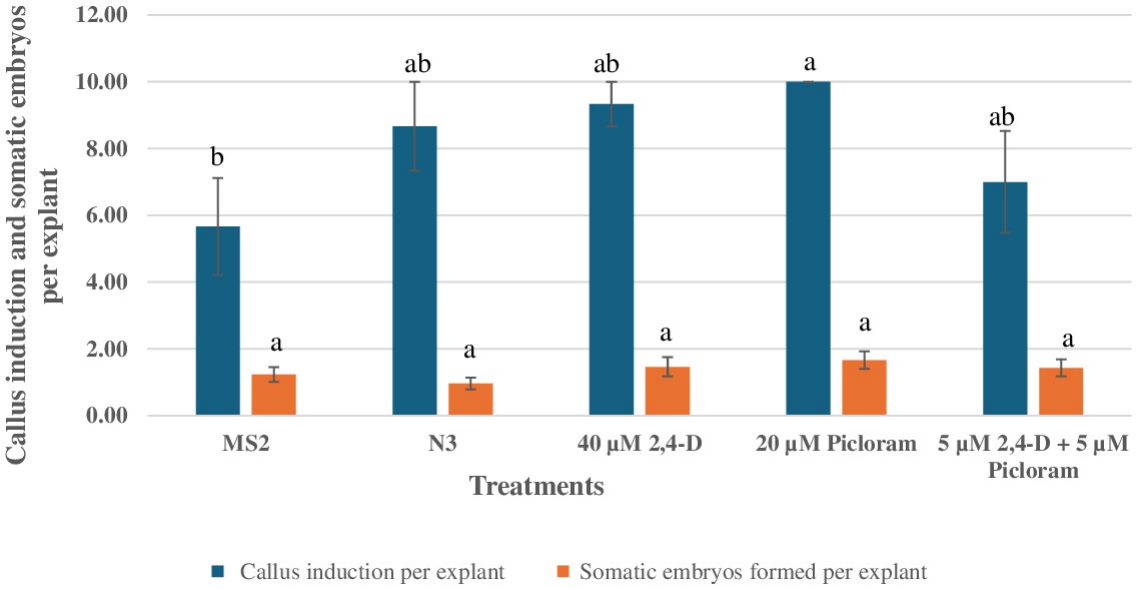
Effect of plant growth regulators on the callus and somatic embryo induction in peanut ‘G-12Y’. Each value represents Mean ± S.E of three replicates. Values with the same superscript are not significantly different at 5% probability level according to DMRT. MS2: 5.0μM BAP + 2.5μM 2,4-D + 5.0μM picloram, 20 g/Lsucrose, pH 6.0; N3: 8.9μM BAP + 4.5μM picloram, 3 g/L phytagel.

Mature embryos explants cultured on various treatments containing BAP, 2,4-D, and picloram in dark, initially swelled and induced yellow friable callus (Fig 1A and 1B) all over the surface in 2 weeks. Callus formation was observed in all the treatments, with differences in both the frequency and characteristics of the callus (Fig 1C). The somatic embryos were formed all over the surface of the explants after three weeks of inoculation in dark (Fig 1D). After 4 weeks in dark, embryogenic callus along with somatic embryos were transferred to PGR free MS medium under 14 h photoperiod for further differentiation and proliferation of somatic embryos (Table 4). Under a dissecting microscope, somatic embryos at various development stages were observed on MS basal medium (Fig 1G–1J). The best response in terms of callus induction and somatic embryogenesis stages (Fig 1E) were observed on MS medium with 20 μM picloram after 7 weeks under 14 h photoperiod. The mean number of callus induction and somatic embryos per explant is provided in Fig 2. There was no statistical difference in the mean number of somatic embryos per explants among treatments, although numerical differences in their frequency were observed.

**Table 4:** Optimized methodology for somatic embryogenesis and shoot induction of peanut cultivar G-12Y.

No.	Stages	Media composition and Procedure	Conditions and Durations	Results
**1**	**Culture establishment**	Seeds were dipped in 70% ethanol for 1 min and embryo was excised. The embryos were immersed in 15% Clorox solution for 15 min and then rinsed three times with distilled water.	All steps were performed under laminar air flow cabinet.	Seeds were surface sterilized. Mature embryos were used as explants.
**2**	**Shoot induction (Direct Organogenesis)**	Shoot induction medium—MS basal medium, TDZ (1,5,10, 15μM), pH 5.8, 7g/L Agar	25 ± 2°C, 14 h light shoot induced (4 weeks) and shoot elongated (8 weeks)	Shoot induced (4 weeks) and elongated (8 weeks) in light on shoot induction and elongation medium.
		Shoot elongation medium- MS basal medium, 5μM BAP, 1μM GA_3,_ pH 5.8, 7g/L Agar		
**3**	**Callus induction**	MS basal medium, 200 mg/L casein hydrolysate, 10 g/L sorbitol, 30 g/L sucrose, picloram (0.1μM– 40.0μM), 2,4-D (0.1μM– 40.0μM),	25 ± 2°C, darkness 4 weeks	After one week in the dark, nodular and friable embryogenic calli were formed, and somatic embryos began to differentiate.
		p H 5.8, 7g/L Agar		
		MS basal medium, 20 g/L sucrose, pH 6.0, 2.5μM BAP + 5.0μM 2,4-D, 5.0μM BAP + 2.5μM 2,4-D +5.0μM picloram, 7g/L agar		
		Nitsch medium, 30 g/L sucrose, pH 5.7, 8.9μM BAP + 4.5μM picloram, 3 g/L phytagel		
**4**	**Stabilization and development of embryogenic callus**	MS basal medium (PGR free MS medium)	25 ± 2°C 14 h light; 7 weeks (2 subcultures).	Embryogenic calli were selected and plenty of embryos were obtained.
**5**	**Somatic embryos germinated into plants**	MS basal medium	25 ± 2°C 14 h light at till plantlet formation.	Conversion of embryos into plantlets. Germinated embryos with well-developed shoots and roots were utilized.
**6**	**Acclimatization of plantlets**	Plants were planted into humidity-controlled boxes with sterile Promix BX.	25 ± 2°C, 16 h light for 21 days until new growth was seen.	Plants were acclimatized.

Fasciated somatic embryos were also reported on PGR free MS medium (Fig 1F) that failed to form well-developed shoots and roots meristems once transferred to MS basal medium under light for germination. Secondary somatic embryogenesis was also evident and formed many somatic embryos (Fig 1K). There was a complete absence of embryogenic callus and somatic embryos on mature embryo explants inoculated on MS Medium with 2,4-D (0.1 and 1.0 μM) and picloram (0.1 and 1.0 μM), individually or in combinations, in dark.

### Cytochemical analysis of embryogenic callus

**Fig 3:**
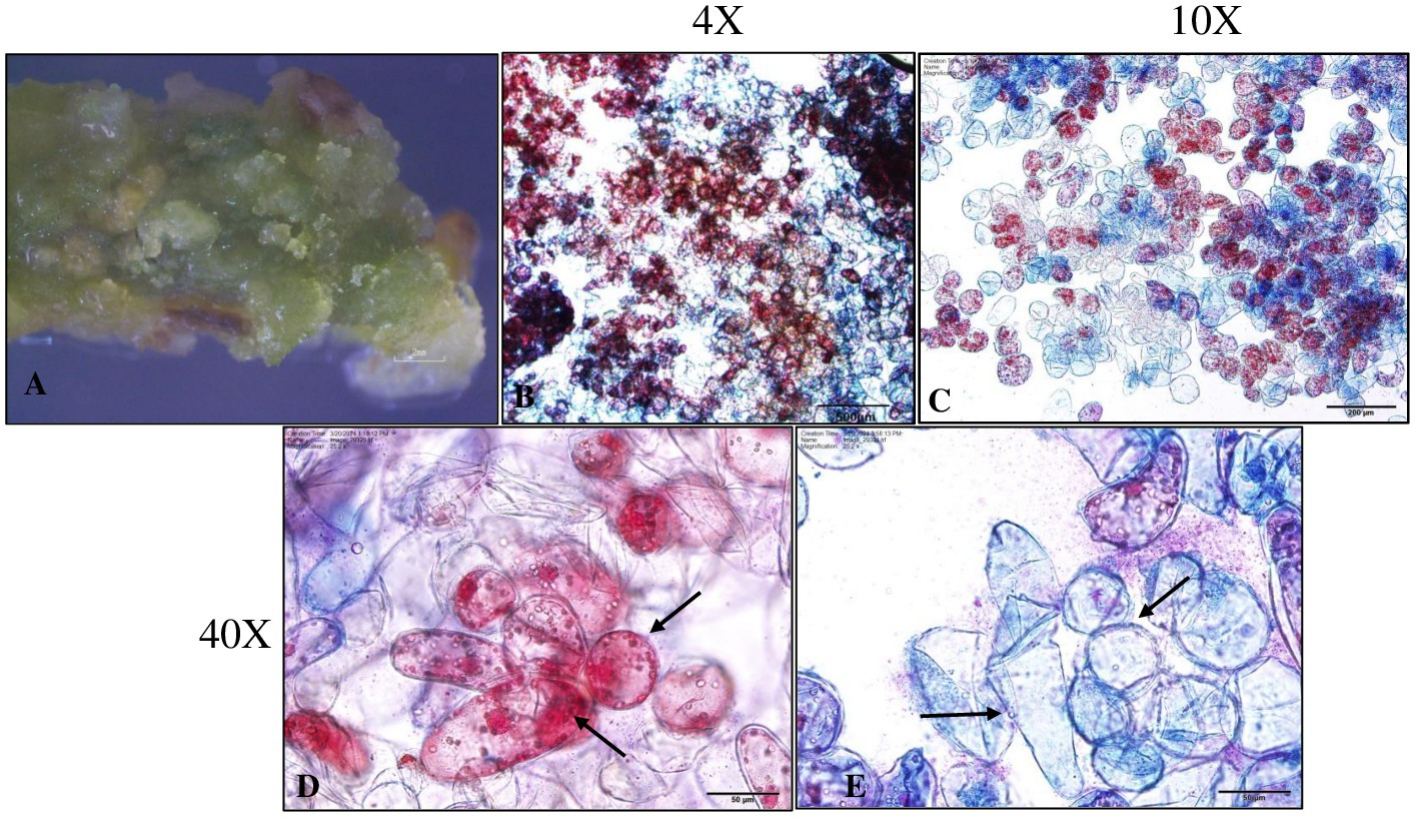
Acetocarmine and Evans blue staining of callus to differentiate embryogenic and non-embryogenic callus in peanut cultivar ‘G-12Y’. (A) Green embryogenic callus on 20 μM picloram in light. (B-C) Embryogenic and non-embryogenic cells are differentiated as red and blue, respectively (4X and 10X respectively). (D) Higher magnification of embryogenic cells (black arrows) with dense cytoplasm (40X). (E) Elongated and vacuolated non-embryogenic cells (black arrows), showing only Evan’s blue stain at higher magnification (40X). Bars: 2 mm (A), 500 μm (B), 200 μm (C), 50 μm (D, E).

Acetocarmine and Evans blue stains were used to differentiate embryogenic (ECs) and non-embryogenic calli (non-ECs). For this, 5-week-old callus cultures under light (Fig 3A) were selected. The embryogenic cells imbibe acetocarmine (red) and are rounded in shape with presence of dense cytoplasm whereas non-embryogenic cells are elongated, vacuolated and permeable to Evans blue (blue) (Fig 3B–3E).

### Somatic embryo development, germination, and plantlet formation

**Fig 4:**
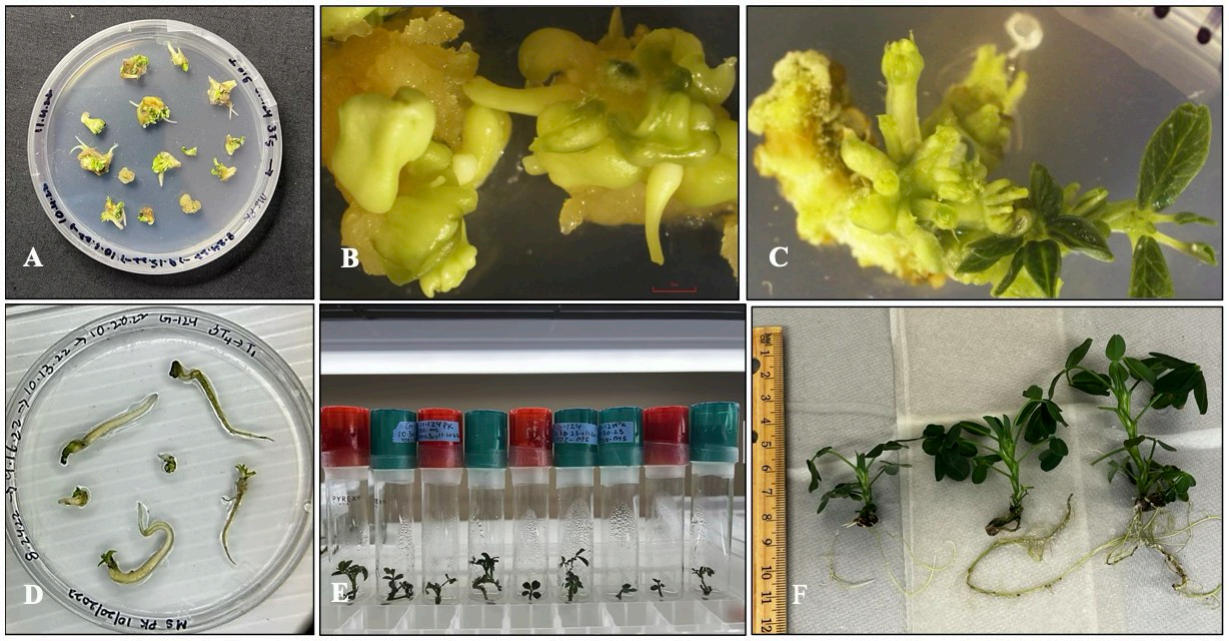
Progressive development of somatic embryos of peanut cultivar ‘G-12Y’. (A) Clusters of somatic embryos in different developmental stages under light after 5 weeks on PGR free MS medium. (B-C) Closer view of asynchronous development of somatic embryos in light after 5 weeks on PGR free MS medium. (D-E) Somatic embryos showing polarity on MS Basal Medium after 7 weeks. (F) Well—Developed shoots and roots formed after 12 weeks on PGR free medium.

**Fig 5:**
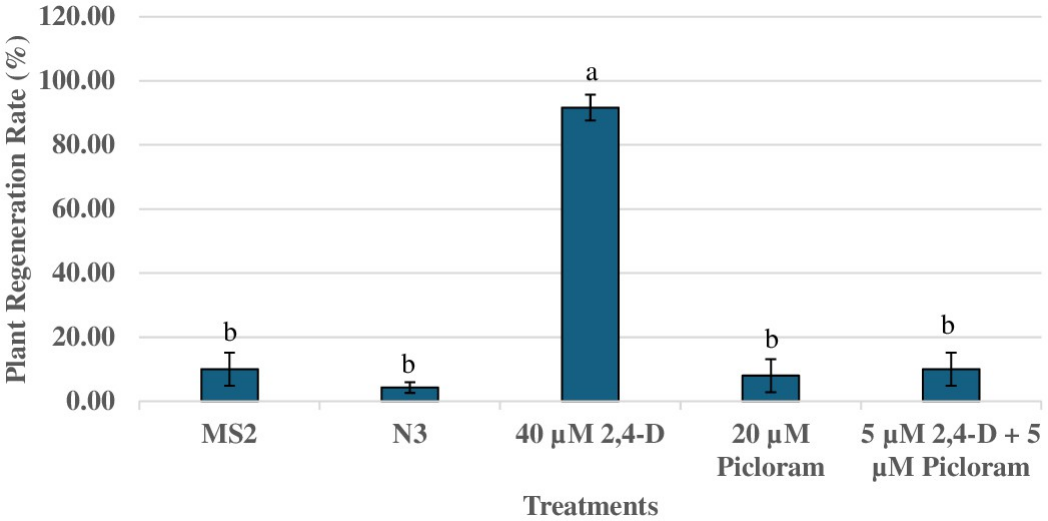
Plant regeneration (%) in peanut G-12Y cultivar from somatic embryos on MS basal medium. Each value represents Mean ± S.E of three replicates. Values with the same superscript are not significantly different at 5% probability level according to DMRT. MS2: 5.0μM BAP + 2.5μM 2,4-D + 5.0μM picloram, 20 g/L sucrose, pH 6.0; N3: 8.9μM BAP + 4.5μM picloram, 3 g/L phytagel.

In the preliminary experiment, germinating embryos were transferred onto various maturation media such as MS medium supplemented with 30 and 20 g/L sucrose, 1μM TDZ, 1μM BAP, 1μM Meta-topolin and MS basal medium for further conversion into plantlets. Among all the maturation media evaluated, MS basal medium responded well for peanut cultivar ‘G-12Y’ (Fig 4). Henceforth, somatic embryos obtained in each treatment were transferred to MS basal medium for maturation and plantlet regeneration. In this study, the somatic embryos developed asynchronously, all stages- globular, heart, torpedo, and cotyledonary were observable in the same medium (Fig 4A–4C). At each subculture on MS basal medium, the embryogenic callus was divided into clumps consisting about 8–9 embryos (Fig 4B). Further, those clumps were separated from each other and sub-cultured again on MS basal medium until plantlet regeneration. The transfer of somatic embryos to MS basal medium changed the morphological appearance and well-developed shoots and roots were formed after 14 weeks (Fig 4D). Upon conversion (development of a shoot and a root), the plantlets were transferred to the test-tubes containing MS basal medium for further growth (Fig 4E and 4F). Among all the treatments, 40 μM 2,4-D induced the highest plantlet regeneration rates (90%) on PGR free MS medium (Fig 5). There were statistically significant differences in plantlet regeneration among various treatments on PGR free MS medium.

### Acclimatization

**Fig 6:**
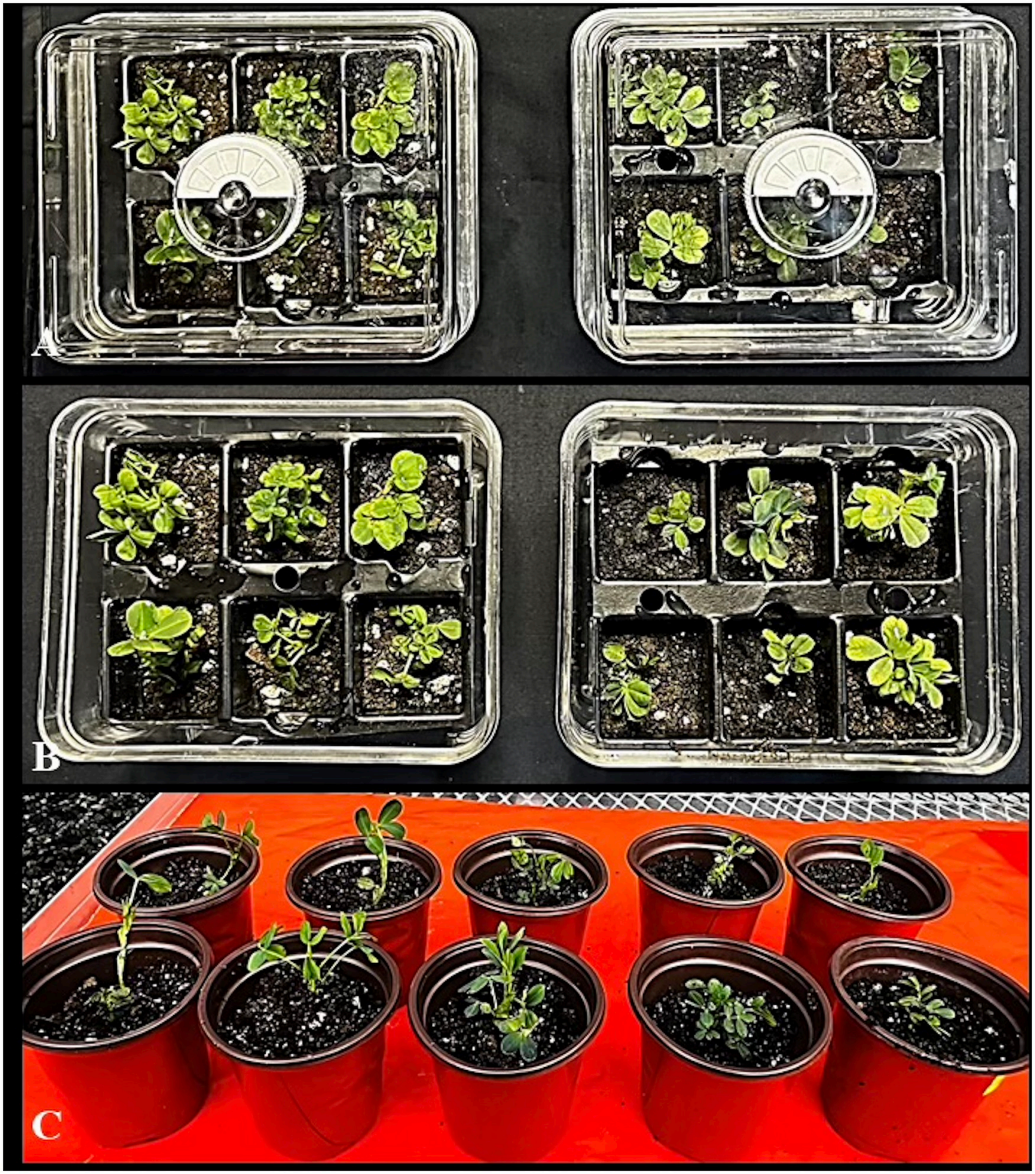
Rooting and plant acclimatization in peanut ‘G-12Y’ well rooted plantlets hardened in sterilized potting mix (Promix BX). (A) Acclimatized plantlets in controlled humid conditions. (B) Acclimatized plantlets in non-humid conditions. Humidity controlled boxes (Smithers- Oasis, Kent, OH, USA). (C) Hardened plantlets showing no abnormalities at 27 ± 2°C with 65–70% relative humidity in the green house.

Plantlets with well-developed shoots and roots, approximately 3 cm in length and bearing 2–3 leaves, were gently washed to remove any residual agar and then transferred to climate-controlled acclimatization boxes. (Smither-Oasis, Kent, OH, USA) containing Promix (BX, Pittsburgh, PA, USA) (Fig 6A). For two weeks, the plantlets were kept in high humidity with the knob (present on top of acclimatized box) closed and then the humidity was reduced gradually by opening the knob periodically for another week (Fig 6B). There were no morphological abnormalities found in the regenerated plantlets of peanut cultivar ‘G-12Y’ showing 100% survival at 65–70% relative humidity and 27 ± 2°C in green house conditions (Fig 6C).

### Histological observation

**Fig 7:**
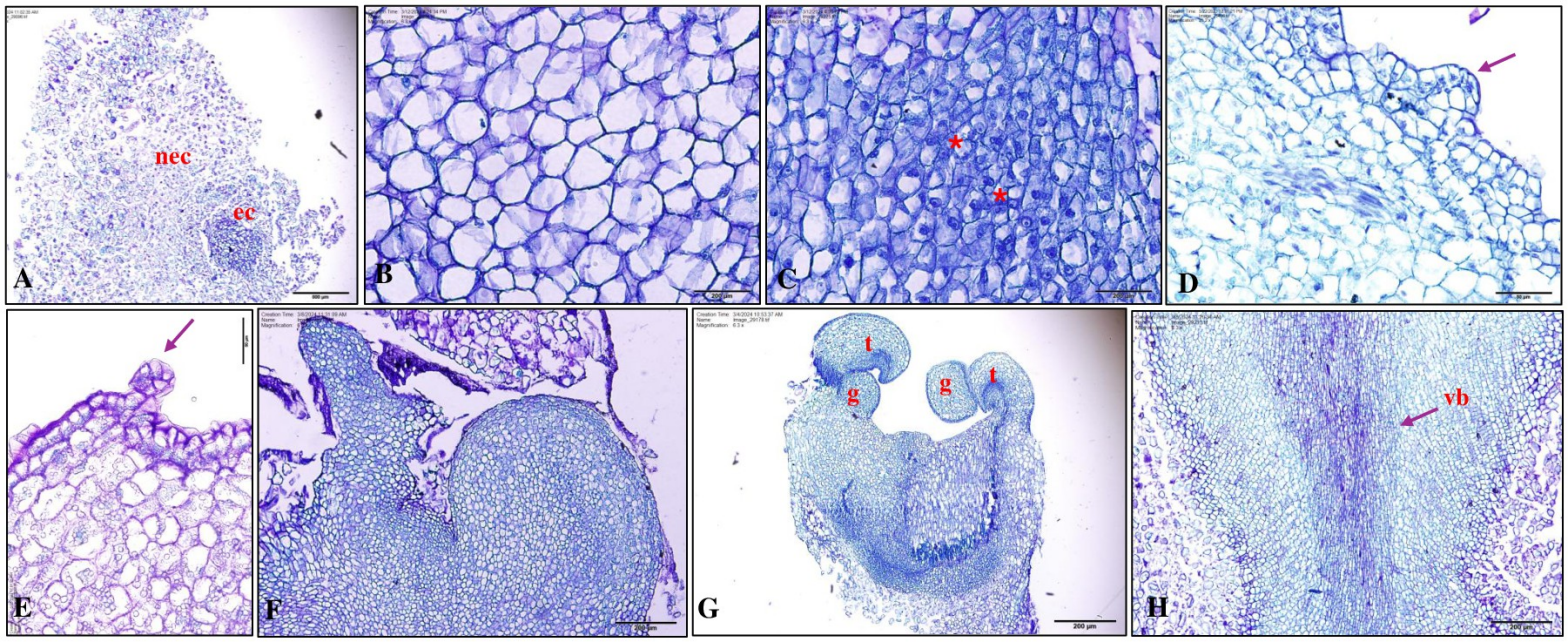
Histological observation of somatic embryogenesis from mature embryo explants of peanut ‘G-12Y’. (A) Embryogenic cells (ec) and non-embryogenic cells (nec) after 1 week of inoculation in dark; darkly stained cell group showing embryogenic cells. (B) Enlarged view of non-embryogenic cells after 1 week of inoculation in dark. (C) Embryogenic cells with thin cell wall and one dividing (*); (D-F) Development of embryoids in 4 weeks (light) callus. (G) Development stages of somatic embryogenesis showing globular (g) and torpedo (t) shaped embryos after 9 weeks in light. (H) Vascular bundle (vb) developed after 9 weeks in light. Bars: A = 500 μm, B-C and F-H = 200 μm, D-E = 50 μm.

Longitudinal paraffin sections (10 μm) of callus and somatic embryos at various developmental stages were cut for histological analysis. Two types of calli were observed after one week of culture in dark conditions (Fig 7A)–one was the non- embryogenic callus composed of elongated cells with little cytoplasm (Fig 7B) and other was embryogenic callus composed of parenchymatous, isodiametric meristematic cells that usually stained dark blue with dense cytoplasm exhibiting dividing cells (Fig 7C). Afterwards, the embryogenic cells continued to differentiate to form globular to various developmental stages after 9-weeks of culture in light condition (Fig 7D–7G). Formation of vascular bundle in somatic embryos was evident as shown in Fig 7H.

### Shoot organogenesis

**Fig 8:**
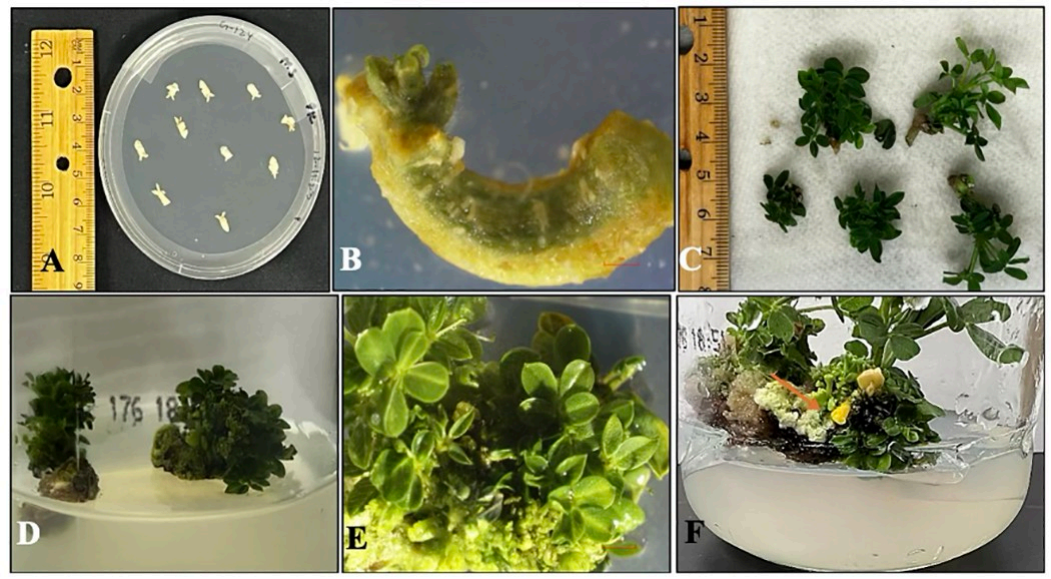
Direct shoot organogenesis on mature embryos explants in peanut ‘G-12Y’. (A) Mature embryos cultured on shoot induction medium (1st Day). (B) Emergence of Shoots on MS medium with 5μM TDZ (light) after 1 week. (C) Multiple shoots were induced on MS medium with 15μM TDZ (light) after 4 weeks. (D) Shoots were proliferated on MS medium with 15μM TDZ (light) after 5 weeks. (E) Closer view of adventitious shoots induced on mature embryo explant. (F) *In-vitro* flowering on MS basal medium after 12 weeks. Bars = 2 mm.

Cytokinins BAP, TDZ and Meta-topolin (1 μM and 5 μM) were tested for shoot induction on mature embryos explants. Thidiazuron (5 μM) supplemented MS medium showed highest shoot induction on mature embryos in comparison to other cytokinins. Following this, another experiment was conducted with 1, 5, 10, and 15 μM of TDZ using mature embryo explants to optimize shoot induction (Table 4, Fig 8A). Direct shoot induction was observed without callus formation after one week incubation under 14 h photoperiod (Fig 8B). Multiple shoots were produced with a maximum of 8–10 shoots per explant after 4 weeks on shoot induction medium (MS medium + 15 μM TDZ. Shoot buds were not developed on the explants incubated on the basal medium devoid of plant growth regulators (Control). Responding explants with shoots were further sub-cultured to shoot elongation medium (MS medium + 5 μM BAP + 1 μM Gibberellic acid) in light conditions after 4 weeks (Table 4) and number of elongated shoots were recorded after 8 weeks on shoot elongation medium (Table 3). It was observed that an increase in TDZ concentration into the MS medium progressively increased the shoot induction and shoot elongation in peanut cultivar ‘G-12Y’. Results suggest that MS + 15 μM TDZ provided highest number of shoot induction being most effective growth regulator type and concentration for direct shoot organogenesis in peanut cultivar ‘G-12Y’ (Table 3, Fig 8C–8E). Later, when shoots induced were sub-cultured on shoot elongation medium, it was observed that MS medium with 5 μM BAP + 1 μM Gibberellic acid (GA_3_) showed good response with 5.40 ± 0.51^a^ (mean number of shoots elongated per explants) after 8 weeks in 14 h photoperiod. Further, subculturing of the shoots on MS basal medium was done to observe further vegetative and reproductive growth of the plants *in-vitro*. It was noted that transferring the shoots to the MS basal medium resulted in *in-vitro* flowering after 12 weeks in 14 h photoperiod (Fig 8F).

## Discussion

### Somatic embryogenesis

Studies on somatic embryogenesis and organogenesis were conducted using mature embryo explants of peanut cultivar ‘Georgia-12Y’. In peanut (*Arachis hypogaea* L.), plant regeneration has been reported earlier via embryogenesis and organogenesis (Table 1). However, despite numerous attempts, peanuts in general are still considered a recalcitrant plant for *in vitro* manipulations [11]. It has been reported that the ability to form somatic embryos is under genetic control and every single genotype within a species can differ in their ability to undergo somatic embryogenesis [30]. Age of explants, explant source, and genotype can affect somatic embryogenesis [31, 32]. While most research studies in peanut focused on immature embryos, hypocotyls, leaves, seedling cotyledons etc., there are a few research studies that investigated mature embryos explants for somatic embryogenesis in *Arachis hypogaea* L. till date (Table 1). Mature zygotic embryo explants offer many benefits such as less contamination rate, by- passing seedling stages and germination and consistent results [33, 34]. Current results are in accordance with Baker et al [12] who reported that embryo axes from mature seed in peanut are efficient and suitable explant for somatic embryogenesis and can be used in a wide range of genotypes. It was found that 20 μM picloram along with casein hydrolysate (0.2 g/L), sucrose (30 g/L) and sorbitol (10 g/L) was very effective to induce somatic embryos in peanut cultivar ‘G-12Y’ in three weeks under dark conditions. In a similar study on *Eucalyptus globulus* [35] 40 μM picloram was more efficient than naphthaleneacetic acid (NAA) to induce somatic embryogenesis. Current results are in alignment with Danso et al. [36] that picloram induced higher number of somatic embryos per callus clump than 2,4-D in cassava (*Manihot esculenta* Crantz) however, picloram required double the concentration of 2,4-D to produce more somatic embryos [36]. Previous peanut researchers often employed extremely high concentrations of auxins to induce somatic embryogenesis. However, in the present study, somatic embryogenesis was successfully achieved using low concentrations of auxins compared to previous studies (Table 1). This approach is crucial because excessively high concentrations can result in somaclonal variations [13]. Further, treatments containing BAP, 2,4-D and picloram on MS and Nitsch and Nitsch media also responded well to induce somatic embryogenesis in peanut cultivar ‘G-12Y’. Current results are in accordance with Nikam et al [37] who suggested the presence of BAP was essential for the induction of embryogenesis in *Agave sisalana*. Effect of casein hydrolysate and sucrose for somatic embryogenesis is in conformity with the results obtained in peanut by Eapen and George [15]. They reported that B5 medium [38] with casein hydrolysate (200 mg/L) and sorbitol (6%) had increased the number of somatic embryos per explant in peanut. In addition, sorbitol can improve the induction of somatic embryos when combined with sucrose by affecting osmotic conditions. It has been shown that sorbitol alone is not effective for somatic embryogenesis, but it can enhance the process when combined with other sugars such as sucrose or mannitol [39]. Higher concentrations of auxins could hinder the development of somatic embryos by disturbing the normal physiological and genetic processes of the cell and may result in abnormality [40]. Therefore, in current study, somatic embryos transferred immediately from induction medium to the MS basal medium after 4 weeks for further development and maturation. Studies also reported that auxins function as an efficient inducer of somatic embryogenesis, but further development of somatic embryos can be achieved by removing or reducing auxin from the medium. It is also known that continuous exposure of explants to high concentration of auxins exogenously interferes with the polar auxin gradient during embryogenesis and prevents the correct apical-basal embryo patterning [41]. PGR- free MS basal medium was found very useful for germination, maturation, and conversion into plantlets in many other plant species such as *Lycium barbarum* L. [25], and *Centella asiatica* L. [42]. Prior report on peanut (Georgia green) using mature embryo-derived leaflets failed to show conversion on MS basal medium and MS + 22.7 μM TDZ. Also, it has been reported that failed somatic embryo conversion to plantlets is linked to plumule malformation or insufficient maturation, which could be genetically regulated [11].

Cell morphology and embryogenic capacity was determined using double staining [28] that was used in the prior study on the somatic embryogenesis in *Lycium barbarum* L. [25]. In addition, histological analysis revealed the cellular organization in embryogenic and non-embryogenic calli along with developmental stages of somatic embryos in peanut cultivar’ Georgia- 12Y’. It was reported earlier that pro-embryogenic masses on the surface or within the callus mass result in the formation of embryogenic callus from which a single cell or clusters of cells develop into somatic embryos [43].

### Shoot organogenesis

Cytokinins are a class of plant hormones that play a crucial role in regulating cell division, differentiation, and growth. They are primarily involved in promoting cell division (cytokinesis) in meristematic tissues, which are regions of actively dividing cells, such as the shoot and root apical meristems [44]. Hence, the study reported various concentrations of TDZ (0.01 μM to 15 μM) to induce adventitious shoot formation from the hypocotyl tissues of peanut (*A*. *hypogaea* cv. EC-5). The current study results are in accordance with the fact that number of shoots increased with increased TDZ concentration [44] but contradicts on using PGR free MS basal medium for further shoot proliferation. However, results from the present study observed shoot elongation on MS medium supplemented with 5 μM BAP + 1 μM GA_3_. Most of the studies reported that combinations of cytokinin or cytokinin with low concentration of auxin results in better response for shoot regeneration. Lamboro et al. [45] found that BAP with TDZ has been effective in promoting shoot induction in peanuts. Medium containing BAP with 2,4-D has also increased the shoot regeneration frequency in peanut [46]. Our results are similar to the observations reported by Gardner [47] that Gibberellic acid (1 μM) may be required to stimulate stem elongation in peanut. It has been reported that Gibberellic acid supports shoot elongation by activating genes involved in growth and promoting the internode elongation [47]. On the contrary Franklin et al. [48] showed that IAA and BAP supplemented with Gibberellic acid resulted in significant reduction in shoot elongation in Pigeon pea.

### *In-vitro* flowering

The present study is the first report of *in-vitro* flowering in peanut cultivar ‘G-12Y’, and these results are in line with [49] that removing of plant growth regulators from the medium has successfully induced *in-vitro* flowering after 12 weeks under 14 h photoperiod. Further optimization to induce *in-vitro* flowering in peanut cultivar ‘G-12Y’ could provide an efficient protocol to induce flowering that can result fertile pegs and complete life cycle under *in-vitro* conditions.

## Conclusions

The current study presents a reproducible, and efficient system for *in-vitro* somatic embryogenesis and organogenesis using mature embryos explants of peanut cultivar ‘G-12Y’. Both types of propagules can be raised simultaneously. This study promises rapid induction of somatic embryos within just three-four weeks in dark conditions. The entire process from maturation, germination to plantlet regeneration in peanut cultivar ‘G-12Y’ needed one medium—PGR free MS basal medium under 16 h photoperiod. Double staining with acetocarmine and Evans blue for the differentiation between embryogenic and non-embryogenic callus was successfully used for the first time in a peanut cultivar. Histological observations confirmed the distinct developmental stages of somatic embryos. It is evident that peanut cultivar ‘Georgia-12Y’ is competent for somatic embryogenesis and shoot organogenesis in the presence of specific plant growth regulators (individually or in combination) and large number of clonal materials can be produced for further improvement through classical breeding or biotechnological interventions. We also optimized a successful acclimatization protocol with an over 95% survival success rate. Another interesting find was the induction of *in vitro* flowering that can specially be helpful to understand unique reproductive biology of peanuts that includes formation of specialized organ ‘peg’ and the seed development. Ongoing efforts are directed to improve somatic embryo numbers and expand optimized protocols to other desirable peanut cultivars.

## Supporting information

S1 File Additional data tables are provided in the supporting information file. (DOCX)

## References

[1] Krapovickas A, Gregory WC. Taxonomia del género Arachis (Leguminosae). Bonplandia. 1994; 8: 1–186. doi: 10.30972/bon.160158

[2] Hassan MU, Akram Z, Ajmal S, Mukhtar T, Nasim S, Shabbir S, et al. Highly efficient *in vitro* root induction in peanut by mechanical stress method. J Anim Plant Sci. 2013; 23: 425–429.

[3] Yang CY, Chen SY, Duan GC. Transgenic peanut (*Arachis hypogaea* L.) expressing the urease subunit B gene of Helicobacter pylori. Curr Microbiol. 2011; 63: 387–391. 21833666 10.1007/s00284-011-9991-4

[4] Khandelwal A, Renukaradhyaa GJ, Rajasekharb M, Sita GL, Shaila MS. Immune responses to hemagglutinin-neuraminidase protein of peste des petits ruminants virus expressed in transgenic peanut plants in sheep. Vet Immunol Immunopathol. 2011; 140: 291–296. doi: 10.1016/j.vetimm.2010.12.007 21211855

[5] Geng L, Niu L, Shu C, Song F, Huang D, Zhang J. High-efficiency regeneration of peanut (*Arachis hypogaea* L.) plants from leaf discs. Afr J Biotech. 2011; 10: 12650–12652.

[6] Quiroz-Figueroa FR, Rojas Herrera R, Galaz-Avalos RM, Loyola-Vargas VM, Embryo production through somatic embryogenesis can be used to study cell differentiation in plants. Plant Cell Tiss Organ Cult. 2006; 86: 258–301. doi: 10.1007/s11240-006-9139-6

[7] Raemakers C, Jacobsen E, Visser RGF. Secondary somatic embryogenesis and applications in plant breeding. Euphytica. 1995; 81: 93–107. doi: 10.1007/BF00022463

[8] Bhatnagar M, Prasad K, Bhatnagar-Mathur P, Narasu ML, Waliyar F, Sharma KK. An efficient method for the production of marker free transgenic plants of peanut (*Arachis hypogaea* L.). Plant Cell Rep. 2010; 29 (5): 495–502. 20217416 10.1007/s00299-010-0838-4

[9] Lakshmanan P, Taji A. Somatic embryogenesis in Leguminous plants. Plant Biol. 1999; 2: 136–148.

[10] George EF. Plant Propagation by Tissue Culture, Part 1: the Technology, Exegetics Limited, Edington. No. Ed. 2, 1993, pp. viii+-574.

[11] Chengalrayan K, Gallo-Meagher M. Evaluation of runner and virginia market types for tissue culture responses. Peanut Sci. 2004; 31: 74–78. doi: 10.3146/pnut.31.2.0003

[12] Baker CM, Durham RE, Burns JA, Parrott WA. High frequency somatic embryogenesis in peanut (*Arachis hypogaea* L.) using mature, dry seed. Plant Cell Rep. 1995;15: 38–42. 24185651 10.1007/BF01690250

[13] Fki L, Masmoudi R, Kriaâ W, Mahjoub A, Sghaier B, Mzid R, et al. Date Palm Micropropagation via Somatic Embryogenesis. Date Palm Biotechnology Springer, Dordrecht. 2011; 47–68. doi: 10.1007/978-94-007-1318-5_4

[14] Xu K, Huang B, Liu K, Qi F, Tan G, Li C, et al. Peanut regeneration by somatic embryogenesis (SE), involving bulbil-like body (BLB), a new type of SE structure. Plant Cell Tissue Organ Cult. 2016; 125:321–328.

[15] Eapen S, George L. Somatic embryogenesis in peanut: influence of growth regulators and sugars. Plant Cell Tiss Organ Cult. 1993; 35 (2): 151–156. doi: 10.1007/BF00037274

[16] Radhakrishnan T, Murthy TGK, Chandran K, Bandyopadhyay A. Micro-propagation in peanut (*Arachis hypogaea* L.). Biol Plant. 2000; 43 (3): 447–450.

[17] Ozias-Akins P, Gill R. Progress in the development of tissue culture and transformation methods applicable to the production of transgenic peanut. Peanut Sci. 2001; 28: 123–131.

[18] Little EL, Magbanua ZV, Parrot WA. A protocol for repetitive somatic embryogenesis from mature peanut epicotyls. Plant Cell Reports. 2000; 19: 351–357. doi: 10.1007/s002990050739 30754786

[19] Venkatachalam P, Geetha N, Khandelwal A, Shaila MS, Sita GL. Agrobacterium-mediated genetic transformation and regeneration of transgenic plants from cotyledon explants of peanut (*Arachis hypogaea* L.) via somatic embryogenesis. Curr Sci. 2000; 78: 1130–1136.

[20] Iqbal MM, Nazir F, Iqbal J, Tehrim S, Zafar Y. *In vitro* micropropagation of peanut (*Arachis hypogaea*) through direct somatic embryogenesis and callus culture. Int J Agric Biol. 2011; 13: 811–814.

[21] Okello DK, Akello LB, Tukamuhabwa P, Ochwo SM, Odong TL, Adriko J, et al. Regeneration procedure for three Arachis hypogaea L. botanicals in Uganda through embryogenesis. Br Biotechnol J. 2015; 7:122–133.

[22] Akasaka YH, Daimon H, Mii M. Improved plant regeneration from cultured leaf segments in peanut (*Arachis hypogaea* L.) by limited exposure to thidia-zuron. Plant Sci. 2000; 156: 169–175. 10936523 10.1016/s0168-9452(00)00251-x

[23] Venkatachalam P, Kavipriya V. Efficient method for in vitro plant regeneration from cotyledonary node explants of peanut (*Arachis hypogaea* L.). Agricultural and Food sciences. 2012; 8–9.

[24] Zhao MX, Qiao LX, Sui JM, Tan LL. An efficient regeneration system for peanut: somatic embryogenesis from embryonic leaflets. J Food Agric Envir. 2012; 10: 527–531.

[25] Khatri P, Joshee N. Effect of picloram and desiccation on the somatic embryogenesis of *Lycium barbarum* L. Plants. 2024 13:151. doi: 10.3390/plants13020151 38256705 PMC10820025

[26] Murashige T, Skoog F. A revised medium for rapid growth and bioassays with tobacco tissue cultures, Physiol. Plant. 1962; 15: 473–497.

[27] Nitsch JP, Nitsch C. Haploid plants from pollen grains, Science. 1969; 163: 85–87. doi: 10.1126/science.163.3862.85 17780179

[28] Gupta PK, Durzan DJ. Biotechnology of somatic polyembryogenesis and plantlet regeneration in loblolly pine. Nat Biotechnol. 1987; 5: 147–151.

[29] Vaidya BN, Jackson CL, Perry Z, Dhekney SA, Joshee N. Agrobacterium-mediated transformation of thin cell layer explants of *Scutellaria ocmulgee* small: a rare plant with anti-tumor properties. Plant Cell Tiss Organ Cult. 2016; 127: 57–69. doi: 10.1007/s11240-016-1029-y

[30] Parrott WA, Merkle WS, Williams EG. Somatic embryogenesis: potential for use in propagation and gene transfer systems. In Murray R.D (ed.), Advanced methods in plant breeding and biotechnology. CAB Intl., Wallingford, UK. 1991. pp. 158–200.

[31] Kundu S, Gantait S. Fundamental Facets of Somatic Embryogenesis and Its Applications for Advancement of Peanut Biotechnology. In: Gosal S., Wani S. (eds) Biotechnologies of Crop Improvement, Volume 1. Springer, Cham. 2018.

[32] Ma HC, McMullen MD, Finer JJ. Identification of a homeobox containing gene with enhanced expression during soybean (*Glycine max* L.) somatic embryo development. Plant Mol Biol. 1994; 24: 465–473. 7907232 10.1007/BF00024114

[33] Tang Y, Liu B, Li XM, Li J, Li HX. Endogenous hormone concentrations in explants and calluses of bitter melon (*Momordica charantia* L.). Interciencia. 2010; 35: 680–683.

[34] McKently AH. Effect of genotype on somatic embryogenesis from axes of mature peanut embryos, Plant Cell Tiss Org Cult. 1995; 42: 251–254.

[35] Corredoira E, Ballester A, Ibarra M, Vieitez AM. Induction of somatic embryogenesis in explants of shoot cultures established from adult Eucalyptus globulus and E. saligna × E. maidenii trees, Tree Physiology. 2015; 35:678–690. doi: 10.1093/treephys/tpv028 25877768

[36] Danso KE, Elegba W, Oduro V. Kpentey PB. Comparative study of 2,4-D and Picloram on friable embryogenic calli and somatic embryos development in cassava (Manihot esculenta Crantz). Int J Integr Biol. 2010; 10: 94–100.

[37] Nikam TD, Bansude GM, Kumar KCA. Somatic embryogenesis in sisal (*Agave sisalana* Perr. Ex. Engelm). Plant Cell Rep. 2003; 22: 188–194. doi: 10.1007/s00299-003-0675-9 12920563

[38] Gamborg O, Miller R, Ojimi K. Nutrient requirements of suspension cultures of soybean root cells, Exp. Cell Res.1968; 50:151–158. doi: 10.1016/0014-4827(68)90403-5 5650857

[39] Canhoto JM, Cruz GS. Improvement of somatic embryogenesis in *feijoa sellowiana* berg (myrtaceae) by manipulation of culture media composition. In Vitro Cell Dev Biol. 1994; 30: 21–25.

[40] Vondrakova Z, Eliasova K, Fischerova L, Vagner M. The role of auxins in somatic embryogenesis of *Abies alba*. Open Life Sci. 2011; 6: 587–596.

[41] Liu CM, Xu ZH, Chua NH. Auxin polar transport is essential for the establishment of bilateral symmetry during early plant embryogenesis. Plant Cell. 1993; 5: 621–30. doi: 10.1105/tpc.5.6.621 12271078 PMC160300

[42] Joshee N, Biswas BK, Yadav AK. Somatic embryogenesis and plant development in *Centella asiatica* L., a highly prized medicinal plant of the tropics. HortScience. 2007; 42: 633–637.

[43] Jim’enez VM, Bangerth F. Endogenous hormone concentrations and embryogenic callus development in wheat. Plant Cell Tiss Org Cult. 2001; 67: 37–46, doi: 10.1023/A:1011671310451

[44] Li Z, Jarret RL, Pittman RN. Shoot organogenesis from cultured seed explants of peanut (*Arachis hypogaea* L.) using thidiazuron. In Vitro Cell Dev Biol- Plant. 1994; 30: 187–191, doi: 10.1007/BF02823030

[45] Lamboro A, Han X, Yang G, Li X, Yao D, Song B, et al. Combination of 6-benzylaminopurine and thidiazuron promotes highly efficient shoot regeneration from cotyledonary node of mature peanut (*Arachis hypogaea* L.) cultivars. Phyton-Int J Exp Bot. 2022; 91:12.

[46] Sharma KK, Anjaiah V. An efficient method for the production of transgenic plants of peanut (*Arachis hypogaea* L.) through Agrobacterium tumefaciens-mediated genetic transformation. Plant Sci. 2000; 159: 7–1. 11011088 10.1016/s0168-9452(00)00294-6

[47] Gardner FP. Growth and partitioning in peanut as influenced by gibberellic acid and daminozide. Agronomy Journal. 1988; 80:159–163.

[48] Franklin G, Jeyachandran R, Ignicimuthu S. Factors affecting regeneration of pigeon pea (*Cajanus cajan* L. Millsp) from mature embryonal axes. Plant Grow Reg. 2000; 30: 31–36.

[49] Faustinelli PC. *In-vitro* Peanut Culture: From seed to seed, Current Protocols. 2023; 3. e918. doi: 10.1002/cpz1.918 37929696

