# Correction: Somatic embryogenesis and shoot organogenesis in peanut cv. ‘Georgia-12Y’ and successful transfer to the soil

**DOI:** 10.1371/journal.pone.0321809

**Published:** 2025-04-01

**Authors:** Poonam Khatri, Nirmal Joshee

There is a typographical error in Table 2. In the sucrose concentration column (column 6), the entry currently reads ‘3.0’, but it should be ‘30’. Additionally, at the bottom of the table, ‘Nitsch & Nitsch’ has been corrected to ‘Nitsch & Nitsch (1969)’. Please refer to the corrected Table 2 here.

**Table 2:** Somatic embryo induction in response to various plant growth regulators in peanut ‘G-12Y’ mature embryo explants.

Treatments	Medium	BAP (µM)	2,4-D (µM)	Picloram (µM)	Sucrose (g/L)	Casein hydroly-sate (g/L)	Sorbitol (g/L)	Gelling agent (g/L)	pH	SE Induction
Control	MS	–	–	–	30	–	–	Agar (7.0)	5.8	No response
1	MS	2.5	5.0	–	20	–	–	Agar (7.0)	6.0	Callus
2	MS	5.0	2.5	5.0	20	–	–	Agar (7.0)	6.0	*Somatic embryos*
3	N&N	8.9	–	4.5	30	–	–	Phytagel (3.0)	5.7	*Somatic embryos*
4	MS	–	20.0	–	30	0.2	–	Agar (7.0)	5.8	Callus
5	MS	–	30.0	–	30	0.2	–	Agar (7.0)	5.8	Callus
6	MS	–	40.0	–	30	0.2	10	Agar (7.0)	5.8	*Somatic embryos*
7	MS	–	–	20.0	30	0.2	10	Agar (7.0)	5.8	*Somatic embryos*
8	MS	–	–	30.0	30	0.2	10	Agar (7.0)	5.8	Callus
9	MS	–	**–**	40.0	30	0.2	10	Agar (7.0)	5.8	Callus
10	MS	–	0.1		30	–	–	Agar (7.0)	5.8	Elongation of embryos
11	MS	–	1.0		30	–	–	Agar (7.0)	5.8	Elongation of embryos
12	MS	–	10.0		30	–	–	Agar (7.0)	5.8	Callus
13	MS	–	–	0.1	30	–	–	Agar (7.0)	5.8	Callus
14	MS	–	–	1.0	30	–	–	Agar (7.0)	5.8	Callus
15	MS	–	–	10.0	30	–	–	Agar (7.0)	5.8	Callus
16	MS	–	0.05	0.05	30	–	–	Agar (7.0)	5.8	Elongation of embryos
17	MS	–	0.5	0.5	30	–	–	Agar (7.0)	5.8	Elongation of embryos
18	MS	–	5.0	5.0	30	–	–	Agar (7.0)	5.8	*Somatic embryos*

SE: Somatic Embryo; N&N: Nitsch and Nitsch (1969)
