# Supplementary material for: Somatic embryogenesis and shoot organogenesis in peanut cv. ‘Georgia-12Y’ and successful transfer to the soil
Source: PLoS One. 2024 Dec 6;19(12):e0315060. doi: 10.1371/journal.pone.0315060 (PMC11623556; doi:10.1371/journal.pone.0315060)
Supplement: S1 File — (DOCX) [file pone.0315060.s001.docx]

**Supplementary Material**

**Somatic Embryogenesis and Shoot Organogenesis in Peanut cv. ‘Georgia-12Y’ and Successful Transfer to the Soil**

^1^Poonam Khatri, and ^1^Nirmal Joshee

^1^Agricultural Research Station, Fort Valley State University, Fort Valley, GA, USA

**Corresponding author**

E-mail: [josheen@fvsu.edu](mailto:josheen@fvsu.edu) (NJ);

+1 478. 822.7039

**S1 Table: The values behind the means, standard deviations and other measures reported for callus induction per explant**

| **Treatments** | **Replications (3)** | **Embryogenic callus per explants** |
| --- | --- | --- |
| **MS2** | 1 | 8 |
|  | 2 | 6 |
|  | 3 | 3 |
| **N3** | 1 | 10 |
|  | 2 | 6 |
|  | 3 | 10 |
| **2,4-D (40µM)** | 1 | 10 |
|  | 2 | 10 |
|  | 3 | 8 |
| **Picloram (20µM)** | 1 | 10 |
|  | 2 | 10 |
|  | 3 | 10 |
| **5µM 2,4-D + 5µM Picloram** | 1 | 5 |
|  | 2 | 10 |
|  | 3 | 6 |

**S2 Table: The values behind the means, standard deviations, and other measures reported for somatic embryos per explant**

| **Treatment** | **Replications** | **Explants numbers** | | | | | | | | | |
| --- | --- | --- | --- | --- | --- | --- | --- | --- | --- | --- | --- |
|  |  | 1 | 2 | 3 | 4 | 5 | 6 | 7 | 8 | 9 | 10 |
| **MS2** | 1 | 1 | 1 | 1 | 1 | 0 | 0 | 0 | 0 | 0 | 0 |
|  | 2 | 4 | 5 | 1 | 1 | 2 | 2 | 1 | 1 | 1 | 2 |
|  | 3 | 1 | 1 | 1 | 1 | 4 | 2 | 1 | 0 | 1 | 1 |
| **N3** | 1 | 2 | 0 | 0 | 1 | 2 | 1 | 3 | 0 | 0 | 1 |
|  | 2 | 1 | 1 | 1 | 1 | 1 | 2 | 2 | 0 | 0 | 3 |
|  | 3 | 0 | 0 | 0 | 2 | 0 | 2 | 2 | 0 | 0 | 1 |
| **2,4-D (40µM)** | 1 | 4 | 5 | 4 | 2 | 2 | 1 | 0 | 0 | 0 | 0 |
|  | 2 | 2 | 2 | 4 | 2 | 1 | 0 | 0 | 0 | 0 | 0 |
|  | 3 | 2 | 2 | 4 | 2 | 1 | 1 | 1 | 1 | 0 | 0 |
| **Picloram (20µM)** | 1 | 4 | 3 | 2 | 2 | 4 | 4 | 0 | 0 | 2 | 2 |
|  | 2 | 2 | 2 | 3 | 2 | 1 | 0 | 0 | 0 | 4 | 4 |
|  | 3 | 1 | 1 | 1 | 1 | 0 | 0 | 0 | 0 | 3 | 2 |
| **5µM 2,4-D+5µM Picloram** | 1 | 2 | 4 | 2 | 1 | 2 | 0 | 0 | 4 | 2 | 0 |
|  | 2 | 0 | 1 | 2 | 2 | 0 | 0 | 0 | 1 | 4 | 0 |
|  | 3 | 3 | 2 | 3 | 0 | 0 | 0 | 0 | 3 | 2 | 3 |

**S3 Table: The values used to build graphs for callus induction and number of somatic embryos per explant**

| Treatments | Callus induction per explant | Number of somatic embryos formed per explant |
| --- | --- | --- |
| MS2 | 5.67 ± 1.45^b^ | 1.23 ± 0.22^a^ |
| N3 | 8.67 ± 1.33^ab^ | 0.97 ± 0.17^a^ |
| 2,4-D (40µM) | 9.33 ± 0.66^ab^ | 1.47 ± 0.28^a^ |
| Picloram (20µM) | 10.00 ± 0.00^a^ | 1.67 ± 0.26^a^ |
| 5µM 2,4-D + 5µM Picloram | 7.00 ± 1.52^ab^ | 1.43 ± 0.25^a^ |

**S4 Table: The values behind the means, standard deviations and other measures reported for plant regeneration (%)**

| **Treatments** | **Replications (Each treatment has five baby food jars containing ten explants), Plant regeneration (%)** | | | | |
| --- | --- | --- | --- | --- | --- |
|  | **1** | **2** | **3** | **4** | **5** |
| **MS2** | 30 | 0 | 0 | 20 | 10 |
| **N3** | 0 | 0 | 10 | 0 | 0 |
| **2,4-D (40µM)** | 100 | 90 | 100 | 80 | 100 |
| **Picloram (20µM)** | 0 | 30 | 0 | 20 | 0 |
| **5µM 2,4-D + 5µM Picloram** | 0 | 10 | 30 | 0 | 20 |

**S5 Table: The values used to build graphs for plant regeneration (%)**

| Treatments | Plant regeneration (%) |
| --- | --- |
| MS2 | 10.00 ± 5.16b |
| N3 | 4.29 ± 1.66b |
| 2,4-D (40µM) | 91.67 ± 4.01a |
| Picloram (20µM) | 8.00 ± 5.16 b |
| 5µM 2,4-D + 5µM Picloram | 10.00 ± 5.16b |
